# Data for the Determination of Total Carbon in Biosolids using MID-Infrared Spectroscopy

**DOI:** 10.1016/j.dib.2020.105615

**Published:** 2020-04-22

**Authors:** N. Albuquerque, B. Meehan, J. Hughes, A. Surapaneni

**Affiliations:** a School of Science. RMIT University, GPO Box 2476, Melbourne 3001, VIC, Melbourne, Australia; b South East Water Corporation, Water's Edge, 101 Wells Street, Frankston 3199, Victoria, Australia

**Keywords:** Biosolids, Sewage Sludge, Total Carbon Analysis, MID-IR, Spectroscopy, Carbon Sequestration

## Abstract

The data presented in this article relates to “Determination of Total Carbon in Biosolids using MID-Infrared Spectroscopy” published in Science of the Total Environment. In this new article, we present the data used for the development of the methodology using Partial Least Squares (PLS) combined with MID-Infrared (MID-IR) spectroscopy for the determination of total carbon in biosolids. Based on the data used, MID-IR combined with PLS was found to be an acceptable alternative and inexpensive method to determine the total C of biosolids compared to conventional methods such as the Dumas combustion method using a LECO C analyser.

Specification Table

**Table:** 

Subject	Analytical Chemistry, Waste Management and MID-IR Spectroscopy
Specific subject area	Biosolids, Sewage Sludge, Total Carbon Analysis, MID-IR, Spectroscopy, Carbon Sequestration.
Type of data	Figures, raw spectral data
How data were acquired	Total carbon acquired using LECO carbon analyser. The MIR-IR spectra were acquired using a PerkinElmer 100 Fourier transform infrared (FT-IR) instrument with a DRIFT attachment. Principal Components Analysis (PCA) was carried out using the Minitab 17 statistical software package. Partial Least Squares modelling was carried out using the Unscrambler 10.5 program produced by CAMO.
Data format	Primary spectral data which consists of absorbances versus wavenumber at 1 wavenumber intervals from 4000 - 400 cm^−1^ (columns) and presented in an Excel spreadsheet for each of 392 samples (rows), and figures showing raw and partially processed spectra and also a schematic diagram of the data processing steps.
Parameters for data collection	Spectral data was generated from 1,900 archived biosolids samples representative of the four types of biosolids produced by wastewater treatment plants in Australia. Samples were specifically selected to cover a broad range of percentage carbon (%C) based on the LECO analyser data in order to develop a PLS model from spectral data for the determination of %C in typical biosolids products.
Description of data collection	Spectra of all biosolids were collected on ball mill-ground samples using diffuse reflectance Fourier transform mid-infrared spectroscopy and accumulating sample data from 64 scans in the 4000-400cm^−1^ range resulting in absorbance data at each wavenumber for each sample. The spectra were mean-centered to eliminate matrix variation effects.
Data source location	RMIT University, Melbourne, Australia
Data accessibility	Raw MID-infra-red spectral data and corresponding total carbon data are available on Mendeley DOI: 10.17632/p64dtjx7rj.1 The remaining supplementary data are available on Mendeley DOI: 10.17632/kbd2g7j7yd.1
Related research article	Albuquerque N, Meehan B, Hughes J, Surapaneni A. Determination of total carbon in biosolids using MID-infrared spectroscopy. Science of The Total Environment 2020; 698: 134195. https://doi.org/10.1016/j.scitotenv.2019.134195

## Value of the data

• The spectral data and corresponding LECO information consists of data that was generated from a large archive of biosolids that are representative of all biosolids products commercially produced in Australia and would, therefore, be of use to scholars interested in biosolids research.
• The data could be used to undertake analysis of MID-IR spectral absorbance data to enable % C analysis of biosolids products generated in other countries.
• The data could be used for data exploration experiments using commercial chemometrics packages in order to evaluate/develop/improve methods to clean biosolids MID-IR spectral data to minimise prediction errors.
• The data could also be used to investigate techniques to reduce prediction errors in the selected wavelength approach in order to identify the spectral information that best represents the C component of biosolids material which could potentially reduce the time required to accumulate spectral data.

## 1 Data Description

**Figure 1:**
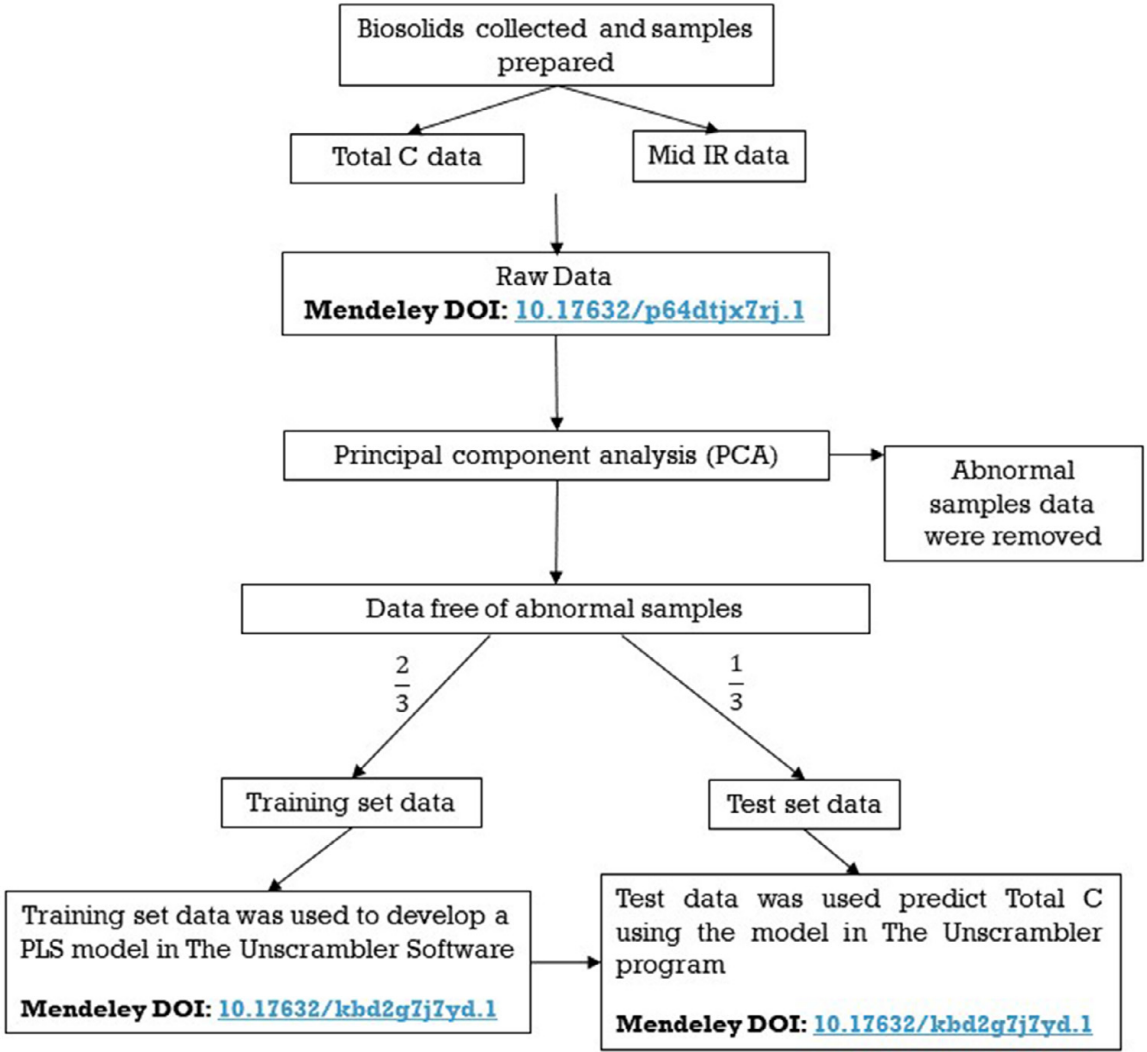
Flow chart showing the raw data processing steps from initial spectra acquisition to the %C result.

The raw data generated by this research is MID-IR spectral absorbance data (with corresponding %C data from LECO analysis) for biosolids products generated from sewage sludges in Australia. The raw data available on Mendeley DOI: 10.17632/p64dtjx7rj.1 consists of 3600 columns and 392 rows in an Excel spreadsheet showing absorbance versus wavenumber data points for 392 samples of biosolds selected from 1900 archived biosolds samples which represent all biosolids products types generated in Australia and also selected to represent a wide range of %C. Fig. 1 presents a schematic diagram that describes how the raw data was processed to provide analytical data for %C in the biosolids samples. This procedure was applied to both approaches- using the full spectrum and using selected regions of the spectrum. The full spectrum method used the entire MID-IR spectrum for the determination of total C (10.17632/kbd2g7j7yd.1). The selected wavelength method (also known of variable selection) used selected sections of the spectrum with high correlation coefficient values (r > 0.5) with total C.

### 1.1 Generation and analysis of the raw data

**Figure 2:**
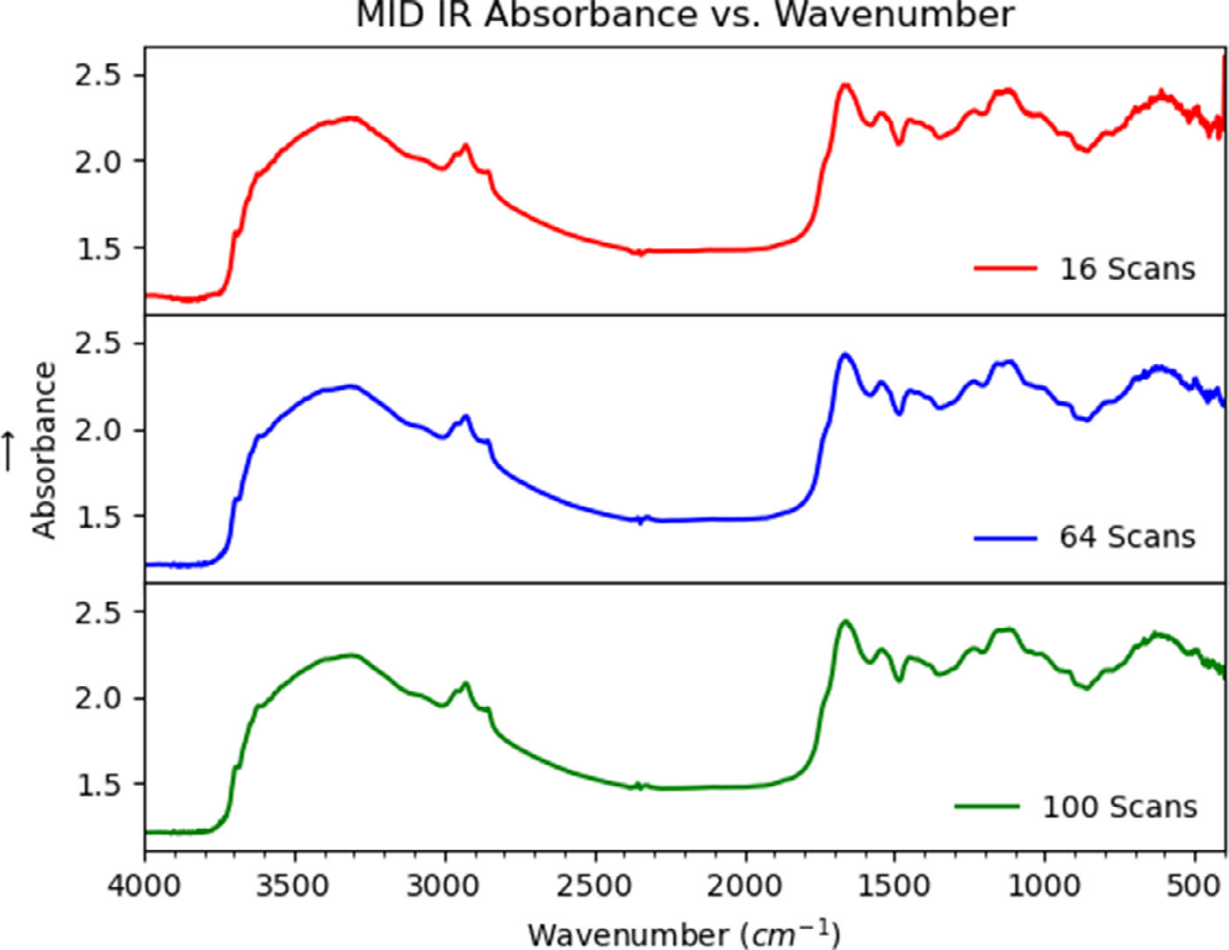
Number of cumulative scans effect on the spectrum.

**Figure 3:**
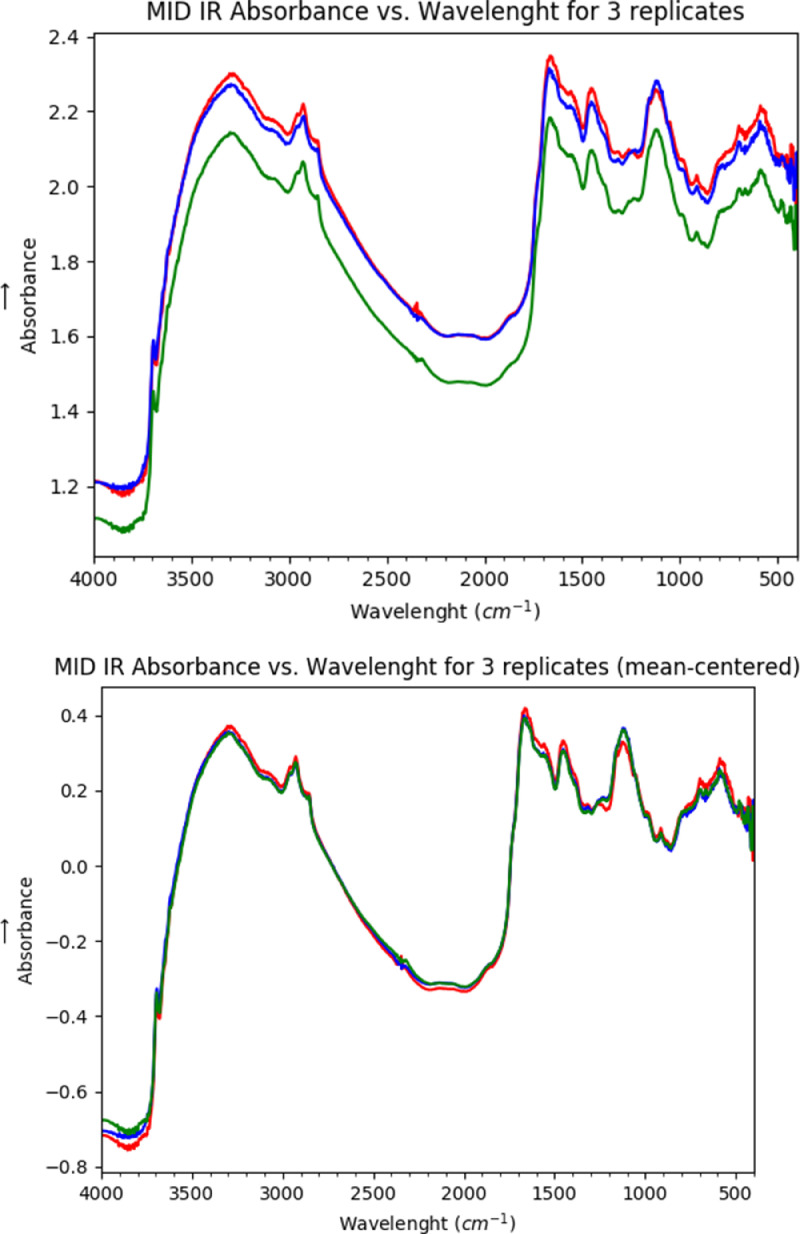
A comparison of non-mean-centred and mean-centred-data.

For each sample, 64 scans were used to generate the data for each MID-IR spectrum (Fig. 2). The MID-IR data and total %C data for 392 biosolids samples contained in the raw data file in the repository (10.17632/p64dtjx7rj.1) were, first mean-centred (Fig. 3) and then analysed using principal components analysis (PCA) and 17 abnormal data points were removed as a first step in the data cleaning process. The data were then randomised and split into two data sets - a training set (n=250) and a test set (n=125). The training set and test set data are contained in the repository (10.17632/kbd2g7j7yd.1). The training set data was used to create the PLS model in The Unscrambler program, and this model was subsequently used to predict total C in the training and test set. The comparison between predicted and actual for the training set and test set are shown in Fig. 2 in Albuquerque et al. [1].

The selected wavelength method was carried out similarly to the full spectrum method. The raw data was mean-centred. The wavelengths regions with correlation coefficient (r) of ≥ 0.5 with total C were selected. These regions were as follows; 1172-1073 cm^−1^, 1575-1551 cm^−1^, 1758-1655 cm^−1^, and 3520-2419 cm^−1^. The remaining wavelength regions were deleted from the data set. The selected wavelength regions were analysed using PCA and 19 abnormal data points were removed (Albuquerque et al. [1]). The remaining samples were separated into a training set (n=249) and a test set (n=124). The training set and the test set data used are provided in the supplementary files. Similar to the full spectral method the training set and test set data was used to create and compare the model, respectively. The comparison between predicted and actual total C for the training set and test set are shown in Fig. 6 in Albuquerque et al. [1].

## 2 Experimental design, materials and methods

A total of 392 biosolids samples were selected from approximately 1900 archived samples which were representative of the four types of biosolids produced by Victorian wastewater treatment plants in Australia. The biosolids samples were analysed for total C, using a LECO TruMac Series C/N analyser, in accordance with standard method 2b described in Rayment and Lyons [2].

The biosolids samples were subsequently analysed by MID-IR in the 4000-400 cm^−1^ range using a PerkinElmer 100 Fourier transform infrared (FT-IR) instrument with a DRIFT attachment. For each sample, 64 scans were used for each MID-IR spectra which were the same number scans used by McCarty et al. [3] and Reeves et al. [4] when analysing soils using MID-IR and near-infrared diffuse reflectance. A detailed description of the methodology can be found in Albuquerque et al. [1].
